# Supplementary material for: Four in every ten infants in Northwest Ethiopia exposed to sub-optimal breastfeeding practice
Source: PLoS One. 2020 Nov 2;15(11):e0238576. doi: 10.1371/journal.pone.0238576 (PMC7605653; doi:10.1371/journal.pone.0238576)
Supplement: Attachment — Submitted filename: Wallenborn_Review.docx [file pone.0238576.s002.docx]

**Four in Every Ten Infants Had Exposed To Inappropriate Breastfeeding Practice Northwest Ethiopia**

Overall a very interesting paper looking at predictors of higher levels of breastfeeding practices in Ethiopia. This study is important since no study has been published in this specific geographic region. However, I think this paper would greatly benefit to some restructuring of the introduction and discussion. I also think the public health significance of this study needs to be further stated. Perhaps with additional analyses where they look at the impact of the breastfeeding index on infant outcomes.

**Abstract**

1. Please add more detail into what is meant by associated factors. Do you mean things that are associated with the index?

**Introduction**

1. “Breastfeeding creates an inimitable psychosocial bond between the mother and baby enhances modest cognitive development and it is the underpinning of the infant’s wellbeing in the first year of life even into the second year of life with appropriate complementary foods from 6 months[1-4].” Very long sentence and it seems like you are trying to say two important things. The benefits of breastfeeding and current recommendations. I would suggest putting these into two separate sentences.
2. You have very good and interesting information in the introduction. However, I think the introduction would benefit from some restructuring. Right now, it seems like the benefits and outcomes of breastfeeding are randomly dispersed throughoug. Instead, make sure to have all benefits in one paragraph, all recommendations in one paragraph, and do this with all major themes. That will help the transition between ideas.

**Methods**

1. To help readers, please state what Kebles are.
2. I am so glad you included how you determined your effect size. I would suggest using the more common terms of power. And then include what you set the main estimators to.
3. For the sampling procedure, it would be good to know if the Kebles were similar in terms of sociodemographic characteristics
4. Important to know how old the children/infants were when the interviews took place. If they were still breastfeeding at the interview, how did you deal with those participants
5. I would suggest making a figure to show exactly how the index was created. Right now, I find it a little confusing and hard to follow. So if you don’t want to make the figure I would suggest making the text a bit more clear. I think this is very important since the index is very interesting and is the major strength of your study
6. Please keep in mind that just because questionnaires were used in previous studies doesn’t mean they provide high data quality. I would add more details into how you considered if these questionnaires provided good data
7. Please also include a list of all confounders you considered
8. Did you get institutional review board approval?

**Results**

1. Is are any outcomes you can look at? To see if the breastfeeding index affects some of the outcomes you discussed in the introduction. This would really strengthen your paper.

**Discussion**

1. The discussion seems a bit long. I would like to see the first paragraph be a general overall and take home messages from your study. 2-3 discussion points for your take home messages. Strengths and limitations section. And finally conclusion with why this matters, and how you can change it..

**Tables**

1. Please create a table showing differences in characteristics between different levels of the breastfeeding index

**Other**

1. I would suggest a different title.
2. I would suggest additional editing to check for general typos
