# Supplementary material for: Four in every ten infants in Northwest Ethiopia exposed to sub-optimal breastfeeding practice
Source: PLoS One. 2020 Nov 2;15(11):e0238576. doi: 10.1371/journal.pone.0238576 (PMC7605653; doi:10.1371/journal.pone.0238576)
Supplement: Attachment — Submitted filename: Rebuttal letter_BFPI.docx [file pone.0238576.s003.docx]

**Rebuttal letter**

**Title:** **FOUR IN EVERY TEN INFANTS HAD EXPOSED TO INAPPROPRIATE BREASTFEEDING PRACTICE IN NORTHWEST ETHIOPIA**

**Ms. Ref. No.:** PONE-D-20-02694

We like to thank the reviewers for the positive and constructive comments with regard to the submission for publication of our manuscript in PLOS ONE**.** We have tried to address all comments and suggestions of the reviewers’ point by point by **writing question number** and **answer** for each and revised our manuscript accordingly. Revisions to the text were highlighted in the manuscript.

**Reviewer #1**

1. Need major improvement in English, suggest to be reviewed by a native medical writer.

**Answer**: Thank you very much for your valuable comments. We have addressed your concerns and made correction.

2. Scoring or index of BFPI needs to be elaborated further, specifically on the different measurement or parameter used, compare to the original source. The adaptation processes of BFPI also need to be explaining exhaustively. Has the questionnaire been validated? Need to indicate if yes or not and explain the reason/argumentation. If yes what is the Cronbach score?

**Answer:** Thank you very much for your valuable comments. We have addressed your concerns and made correction. The questionnaire has been using to measure breast feeding practices in Ethiopia. The pervious study was validated the breast feeding performance index questionnaire in Ethiopia. For this study, we used the already validated tools.

3. Important to briefly describe 'what is principal component analysis (PCA) is?' and the rational of using it is still also missing.

**Answer**: Thank you very much for your valuable comments. We have addressed your concerns and made correction.
4. The occupational factors and other work related determinants should also be explained in detail, to draw a line with the breastfeeding performance index, even though the percentages of the subject who are worker are only around 20%.
**Answer:** Thank you very much for your valuable comments. We have addressed your concerns and made correction.

**Reviewer #2:**

Comment: Overall a very interesting paper looking at predictors of higher levels of breastfeeding practices in Ethiopia. This study is important since no study has been published in this specific geographic region. However, I think this paper would greatly benefit to some restructuring of the introduction and discussion. I also think the public health significance of this study needs to be further stated. Perhaps with additional analyses where they look at the impact of the breastfeeding index on infant outcomes.

**Answer:** Thank you very much for your valuable comments. We have addressed your concerns and made correction.
**Abstract**

1. Please add more detail into what is meant by associated factors. Do you mean things that are associated with the index?

**Answer**: Thank you very much for your valuable comments. We have addressed your concerns and made correction.
**Introduction**

1. “Breastfeeding creates an inimitable psychosocial bond between the mother and baby enhances modest cognitive development and it is the underpinning of the infant’s wellbeing in the first year of life even into the second year of life with appropriate complementary foods from 6 months[1-4].” Very long sentence and it seems like you are trying to say two important things. The benefits of breastfeeding and current recommendations. I would suggest putting these into two separate sentences.

**Answer:** Thank you very much for your valuable comments. We have addressed your concerns and made correction.
2. You have very good and interesting information in the introduction. However, I think the introduction would benefit from some restructuring. Right now, it seems like the benefits and outcomes of breastfeeding are randomly dispersed throughoug. Instead, make sure to have all benefits in one paragraph, all recommendations in one paragraph, and do this with all major themes. That will help the transition between ideas.

**Answer:** Thank you very much for your valuable comments. We have addressed your concerns and made correction.
**Methods**
1. To help readers, please state what Kebeles are.

**Answe**r: Thank you very much for your valuable comments. We have addressed your concerns and made correction.
2. I am so glad you included how you determined your effect size. I would suggest using the more common terms of power. And then include what you set the main estimators to. **Answer:** Thank you very much for your valuable comments. For this study we use prevalence to estimate the sample size. The study was single proportion, not comparison study. We tried to calculate the sample size by considering important factors, but, the calculated sample size was lower than the sample size estimated using prevalence. Finally, we decision to use the large sample size to increase the power of analysis and representative
3. For the sampling procedure, it would be good to know if the Kebeles were similar in terms of socio-demographic characteristics

**Answer:** Thank you very much for your valuable comments. We have addressed your concerns and made correction.
4. Important to know how old the children/infants were when the interviews took place. If they were still breastfeeding at the interview, how did you deal with those participants

**Answer:** Thank you very much for your valuable comments. We have addressed your concerns and made correction.
5. I would suggest making a figure to show exactly how the index was created. Right now, I find it a little confusing and hard to follow. So if you don’t want to make the figure I would suggest making the text a bit more clear. I think this is very important since the index is very interesting and is the major strength of your study

Answer: Thank you very much for your valuable comments. We have addressed your concerns and made correction.
6. Please keep in mind that just because questionnaires were used in previous studies doesn’t mean they provide high data quality. I would add more details into how you considered if these questionnaires provided good data

Answer: Thank you very much for your valuable comments. We have addressed your concerns and made correction.
7. Please also include a list of all confounders you considered

Answer: Thank you very much for your valuable comments. We have addressed your concerns and made correction.
8. Did you get institutional review board approval?

Answer: Thank you very much for your valuable comments. We have addressed your concerns and made correction. The institutional review board approval protocol placed under declaration at end
Results
1. Is/ are any outcomes you can look at? To see if the breastfeeding index affects some of the outcomes you discussed in the introduction. This would really strengthen your paper.

Answer: Thank you very much for your valuable comments. But, we did not included variables which helps to measure the effect of breastfeeding index affects

Discussion
1. The discussion seems a bit long. I would like to see the first paragraph be a general overall and take home messages from your study. 2-3 discussion points for your take home messages. Strengths and limitations section. And finally conclusion with why this matters, and how you can change it.

Answer: Thank you very much for your valuable comments. We have addressed your concerns and made correction.

**Tables**
1. Please create a table showing differences in characteristics between different levels of the breastfeeding index

Answer: Thank you very much for your valuable comments. We have addressed your concerns and made correction.
 **Other**
1. I would suggest additional editing to check for general typos

Answer: Thank you very much for your valuable comments. We have addressed your concerns and made correction.
