# Supplementary material for: Four in every ten infants in Northwest Ethiopia exposed to sub-optimal breastfeeding practice
Source: PLoS One. 2020 Nov 2;15(11):e0238576. doi: 10.1371/journal.pone.0238576 (PMC7605653; doi:10.1371/journal.pone.0238576)
Supplement: Attachment — Submitted filename: Rebuttal letter_BFPI final 12.docx [file pone.0238576.s004.docx]

**Rebuttal letter**

**Title:** **FOUR IN EVERY TEN INFANTS HAD EXPOSED TO INAPPROPRIATE BREASTFEEDING PRACTICE IN NORTHWEST ETHIOPIA**

**Ms. Ref. No.:** PONE-D-20-02694

We have tried to address all comments and suggestions of the ACADEMIC EDITOR/ Editor Comments point by point by revised our manuscript accordingly. Revisions to the text were highlighted in the manuscript.

**ACADEMIC EDITOR/ Editor Comments:** Please restructure the discussion so that you compare your findings with previous literature in one paragraph; followed by two paragraphs providing an explanation into your results. Right now, you have the majority of information you need, there just needs to be some restructuring of paragraphs 2-7 of the discussion.

**Answer**: Thank you very much for your valuable comments. We have addressed your concerns and restructure the discussion part.
